# Air pollution and meteorological conditions significantly contribute to the worsening of allergic conjunctivitis: a regional 20-city, 5-year study in Northeast China

**DOI:** 10.1038/s41377-021-00630-6

**Published:** 2021-09-17

**Authors:** Cheng-Wei Lu, Jing Fu, Xiu-Fen Liu, Wei-Wei Chen, Ji-Long Hao, Xiao-Lan Li, Om Prakash Pant

**Affiliations:** 1 The Department of Ophthalmology, The First Hospital of Jilin University, Changchun, 130021 China; 2 Key Laboratory of Wetland Ecology and Environment, Northeast Institute of Geography and Agroecology, Chinese Academy of Sciences, Changchun, 130102 China; 3 Institute of Atmospheric Environment, China Meteorological Administration, Shenyang, 110166 China; 4 Dhangadhi Netralaya Eye Hospital, Dhangadhi, 3 Kailali Nepal

**Keywords:** Other photonics, Optics and photonics

## Abstract

This study is the first to explore the potential associations among allergic conjunctivitis (AC), air pollution, and meteorological conditions in Northeast China. Data of meteorology, ambient atmospheric pollutants, and the incidence of allergic conjunctivitis (IAC) in prefecture-level cities between the years 2014 and 2018 are analyzed. The results show an increasing trend in the AC of average growth rate per annum 7.6%, with the highest incidence in the provincial capitals. The IAC is positively correlated with atmospheric pollutants (i.e., PM_2.5_, PM_10_, CO, SO_2_, NO_2_, and O_3_) and meteorological factors (i.e., air temperature and wind speed), but negatively correlated with relative humidity. These results suggest that the IAC is directly proportional to pollution level and climatic conditions, and also the precedence of air pollution. We have further obtained the threshold values of atmospheric pollutants concentration and meteorological factors, a turning point above which more AC may be induced. Compared with the air quality standard advised by China and the World Health Organization (WHO), both thresholds of PM_10_ (70 μg m^−3^) and PM_2.5_ (45 μg m^−3^) are higher than current standards and pose a less environmental risk for the IAC. SO_2_ threshold (23 μg m^−3^) is comparable to the WHO standard and significantly lower than that of China’s, indicating greater environmental risks in China. Both thresholds of NO_2_ (27 μg m^−3^) and O_3_ (88 μg m^−3^) are below current standards, indicating that they are major environmental risk factors for the IAC. Our findings highlight the importance of atmospheric environmental protection and reference for health-based amendment.

## Introduction

With the rapid development of industrialization and urbanization, an increasing number of people are now suffering from the adverse effects of increased air quality problems. Globally, air pollution is particularly becoming a main source of mortality and morbidity for human beings. Public health issues, especially allergic diseases, are increasing rapidly^1^. Conjunctiva is an important part of the ocular surface directly exposed to the atmosphere. Allergic conjunctivitis (AC) is a common ocular surface disease that causes dry eye, itching, burning sensation, and may lead to sight-threatening conditions, significantly causing limited physical activity, reductions in work performance, educational productivity, and altering patients’ overall quality of life. It can also inflict enormous economic costs which need to be addressed in welfare and health policies.

Increasing evidence had shown that air pollution or climate change is considered an important risk factor for the development of AC. It not only aggravates the symptoms of AC but also increases the incidence of its severe forms^2^. Some studies have evaluated the effects of air pollution on the AC globally. The prevalence of AC is 40% in the USA^3^, 30% in Japan^4^, 19.2% in Pakistan^5^, and 11.1% in Ethiopia^6^. The overall prevalence of AC among school children in Shanghai, a modern city in East China, is 28%^7^. Meanwhile, the prevalence of AC in Tibet, an undeveloped region in West China, is only 5.2%, much lower than that of Shanghai^8^. The exact prevalence of AC varies in different countries and even in different regions of the same country. Regional environmental risk factors (including air pollution and climatic variations), ethnic differences, heterogeneous nature of the diseases, and allergen species are presumed to be behind these variations. In addition, AC has seasonal variations to which climatic factors may have made the contribution. High temperature, low humidity, and low latitudinal location are factors significantly associated with a high prevalence of AC^9^. It is found that AC had increased incidence in May, September and the valley in winter based on a nationwide, population‐based, cross‐sectional study conducted in Korea^10^. It was speculated that a large temperature difference between the day and night may result in an increase of aeroallergens such as pollen, resulting in AC reactions^10^. Considering AC’s adverse impact on health care, the possible epidemiological exacerbating factors associated with AC should be analyzed at the regional and global level. However, even though a few studies have been conducted to that effect, there are still many countries that remain un-surveyed.

China has been suffered severe air pollution and climate change in the past decades^11^. Previous study by Hong et al. has found that outpatient visits for AC significantly correlate with increased levels of temperature, O_3_, and NO_2_ in Shanghai, China, indicating ambient pollution and weather changes contribute to the onset, as well as the worsening of the condition^12^. In general, ambient air pollution contains microparticles and gaseous pollution. Microparticle pollutants include diesel exhaust particles (DEPs) and particulate matters (PMs). Gaseous pollutants include CO, O_3_, NO_2_, SO_2_, and oxidants. Furthermore, PMs can be classified into two subgroups based on their diameters: PM_2.5_ (<2.5 μm) and PM_10_ (<10 μm). The PM_10_ mainly include mold, dust, and pollen, while PM_2.5_ mainly includes organic compounds, metallic particles, and combustion particles. The DEPs are tinny PMs with a diameter lesser than 0.1 μm. The major source of air pollution is traffic-related air pollutants (TRAPs) in big and crowded cities. TRAPs are a mixture of combustion-derived PMs, DEPs, and gaseous emissions (e.g., NO_2_, CO, oxidants, and organic aerosols). In China, Hong’s study^12^ is the single study done in Southeast China and no study has been done in Northeast China to date.

Being a region with high latitude, Northeast China covers a land area of 787,300 square kilometers and suffers pollution characteristics typical to China. In the past 50 years, the average number of haze days in Northeast China has increased significantly^13^. Sometimes referred to as China’s Rust Belt, it is comparable to its American namesake as it is both a traditional old industrial base, and an important agricultural area. Meanwhile it also has the added complication of being an area, which relies on centrally distributed heating supply in winter. All these resulted in diversified air pollution contributors in Northeast China^14^. The latest studies have suggested burning of agricultural straw has become one of the prominent sources of air pollution^13^, causing frequent seasonal haze events during late autumn or early spring with the highest hourly PM_2.5_ concentrations higher than 1000 μg m^−3^. In addition, Northeast China is surrounded by three mountain ranges on most sides, which is not favorable to the diffusion of air pollution. However, up to date, no studies have been conducted of the relative impact of air pollutants and meteorological variables on AC cases in Northeast China.

In this study, the incidence of allergic conjunctivitis (IAC), air pollutant data, and meteorological data at major cities in the last five years are collected and analyzed to determine whether the prevalence of AC is significantly associated with the air pollution or meteorology conditions. To our knowledge, it is the first study to elucidate the underlying impact of air pollutants and climate factors on AC in Northeast China. We believe it can be of aid in a wiser allocation of stretched resources for AC control and prevention.

## Results

### Spatiotemporality of allergic conjunctivitis

**Fig. 1:**
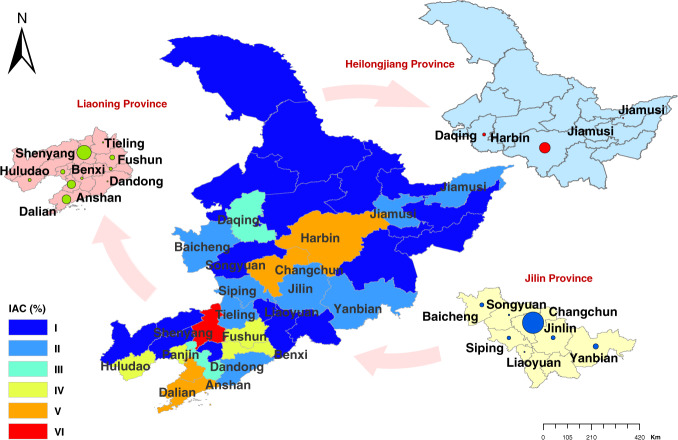
Spatial distribution of the IAC at prefecture-level cities in Northeast China in 2017. The gradient six colors represent the severity of IAC on a scale from I to VI. (i.e., I: 0–0.5%; II: 0.5–2%; III: 2–5%; IV: 5–15%; V: 15–30% and VI: 30–100%)

On provincial scale, the spatiality of IAC showed a prevalence of AC in Liaoning Province, which has a higher IAC as compared to other provinces (Fig. 1). On the city scale, provincial capital cites (i.e., Shenyang, Changchun, and Harbin) have a significantly higher percentage with typical representative characteristics than other cities. The IAC in most other prefecture-level cities is relatively consistent with slight variations. However, significant differences in IAC were determined in cities of Liaoning province, among which Dalian and Anshan, two cities with larger area and population, have higher IAC than most other cities.

**Fig. 2:**
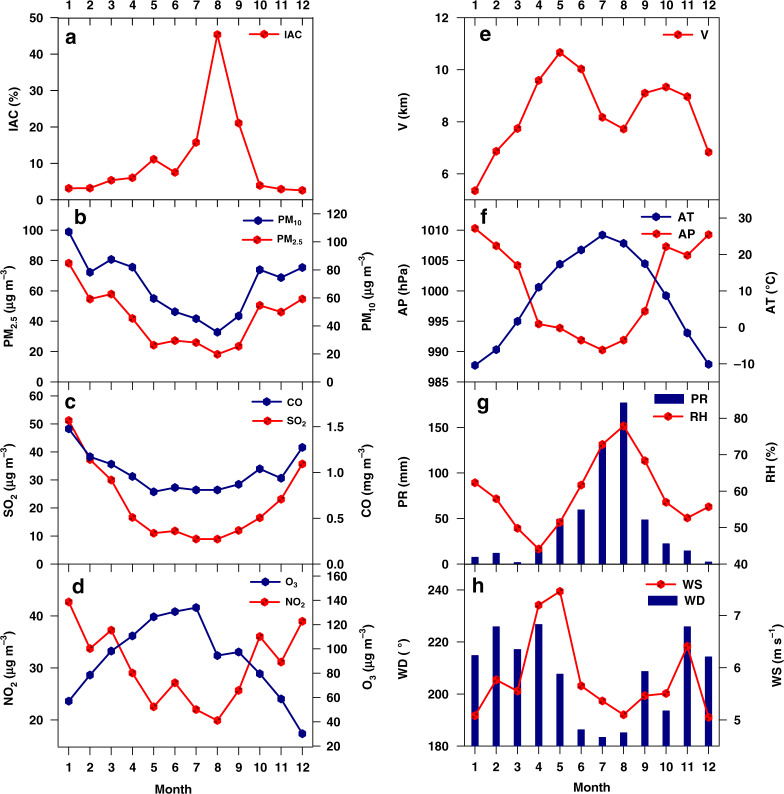
Monthly variations of the IAC. **a**, concentrations of atmospheric pollutants **b**–**d**, and meteorological factors **e**–**h**. The data of three types in each month were averaged by valid data at prefecture-level cities in 2017. IAC incidence of allergic conjunctivitis, AP Air pressure, AT Air temperature, RH Relative humidity, V Visibility, PR Precipitation, WS Wind speed, WD Wind direction

On the interannual scale, the IAC seems to follow a significant increasing trend during 2014–2018 with an average of 7.6% in the three provinces (Table 1). The prevalence increase of Jilin Province is 16.7%, followed by Liaoning (5.7%), and Heilongjiang (5.9%). In addition, monthly variations show higher IAC in late summer and early autumn (i.e., from July to September) with the peak occurring in August (Fig. 2).

**Table 1:** Summary of IAC, atmospheric pollutants (i.e., PM_2.5_, PM_10_, SO_2_, CO, NO_2_, and O_3_) and meteorological factors (i.e., Air pressure, Air temperature, Relative humidity, Visibility, Precipitation, Wind speed, and Wind direction) in Northeast China from 2014 to 2018

Year	Province	IAC(%)	PM_2.5_ (μg m^−3^)	PM_10_ (μg m^−3^)	SO_2_ (μg m^−3^)	CO (mg m^−3^)	NO_2_ (μg m^−3^)	O_3_ (μg m^−3^)	AP (hPa)	AT (°C)	RH (%)	V (km)	PR (mm)	WS_1_ (m s^−1^)	WS_2_ (m s^−1^)	WD (°)
2014	Heilongjiang	10	46	70	23	0.84	34	74	1001	5	63	13.9	2840	2.1	4.6	204
	Jilin	15	45	74	19	0.88	27	88	990	7	60	10.7	525	2.5	5.7	224
	Liaoning	44	47	80	32	1.14	33	93	1009	10	60	9.3	473	2.6	5.4	197
2015	Heilongjiang	12	44	65	19	0.82	31	75	1000	6	64	12.1	2737	2.2	4.8	–
	Jilin	20	45	74	20	0.87	27	88	990	7	62	6.7	522	2.7	5.6	–
	Liaoning	40	47	80	32	1.14	33	93	1009	10	63	8.7	595	2.7	5.5	–
2016	Heilongjiang	15	40	61	16	0.79	29	78	999	9	66	5.2	580	2.4	5.2	192
	Jilin	23	45	73	20	0.87	28	87	988	10	65	6.6	744	2.8	6.0	211
	Liaoning	43	47	80	30	1.16	32	92	1009	10	63	7.9	783	2.7	5.7	190
2017	Heilongjiang	20	39	59	15	0.75	27	79	1000	5	62	7.4	505	2.2	5.1	209
	Jilin	25	44	72	19	0.88	28	85	989	7	60	6.8	533	2.7	6.0	223
	Liaoning	62	46	79	30	1.16	32	93	1009	10	59	9.7	562	2.6	5.7	195
2018	Heilongjiang	10	35	55	13	0.69	25	79	1000	5	63	7.5	626	–	5.0	201
	Jilin	28	42	70	19	0.86	28	83	989	7	61	7.0	611	–	5.9	218
	Liaoning	51	46	78	29	1.15	32	93	1009	10	60	11.1	590	–	5.7	185

IAC represents the relative proportion of the incidence of allergic conjunctivitis; AP Air pressure, AT Air temperature, RH Relative humidity, V Visibility, PR Precipitation, WS Wind speed, WD Wind direction; WS_1_ and WS_2_ represent wind speed in the height of 2 m and 10 m, respectively. The data of each province is averaged by the cities with valid data of IAC.

### Spatiotemporality of air pollution and meteorological factors

**Fig. 3:**
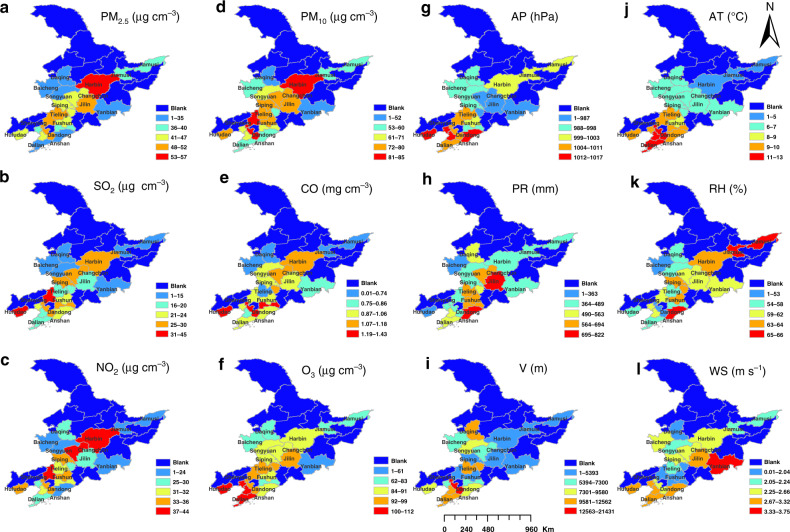
Spatial distributions of annual concentrations of atmospheric pollutants. **a**–**f** and annual average or cumulative values (i.e., precipitation) of meteorological factors **g–l** in 2017. AP Air pressure, AT Air temperature, RH Relative humidity, V Visibility, PR Precipitation, WS Wind speed

Spatially, annual concentrations of particulate matters (i.e., PM_10_ and PM_2.5_) and most gaseous pollutants (i.e., SO_2_, NO_x_, and CO) are highest in provincial capital cities, especially for Jilin province and Heilongjiang province (Fig. 3), indicating strong emission. For O_3_, maximum values are observed in Jilin city of Jilin province, and high levels of O_3_ concentrations are found in coastal cities (i.e., Huludao, Panjin, Jinzhou, and Dalian) of Liaoning province. Temporally, the interannual data shows that the annual average concentration of O_3_ shows an increasing trend during the observation period, while the concentration of other atmospheric pollutants (i.e., PM_10_, PM_2.5_, SO_2_, NO_x_, and CO) exhibits a decreasing trend, fluctuating from year to year. In general, higher concentrations of most atmospheric pollutants (i.e., PM_10_, PM_2.5_, SO_2_, NO_x_, and CO) occur between October and the following March with two peaks occurring in October and January, which are closely linked to intensities of coal combustion for heating supply and large-scale open-air burning of crop straw. The monthly trend of O_3_ concentrations is the opposite of that of other atmospheric pollutants, with the maximum occurring in June or July and the minimum in January or December. This pattern is related to the formation mechanism of O_3_, with the highest light intensity and volatile organic compound (VOC) emissions from natural sources in summer.

The monthly variation of visibility presents double peaks of May and September or October, and the valley value is in July or August, which is mainly the result of intense rainfall during the two months (Fig. 2). For air temperature and pressure, there is the reverse unimodal trend. The air pressure was at its lowest in July when the temperature is at its highest, and it is at its highest in January when the temperature is at its lowest. The maximum relative humidity appears in August, when rainfall is at its highest level, and the minimum in April, the driest month. In addition, the wind speed and direction both demonstrate strong seasonal characteristics. The wind is strongest in April and May, then in November, and wind direction is mainly southwest. During 2014–2018, meteorological factors did not have significant changes year on year at provincial scale, which indicates the long-term nature of climate change. Moreover, the spatial distribution of urban meteorological factors varied greatly in Northeast China, which we believe are influenced by climate zone, topography, land use, and urban construction.

### Correlation of allergic conjunctivitis with environmental factors

**Fig. 4:**
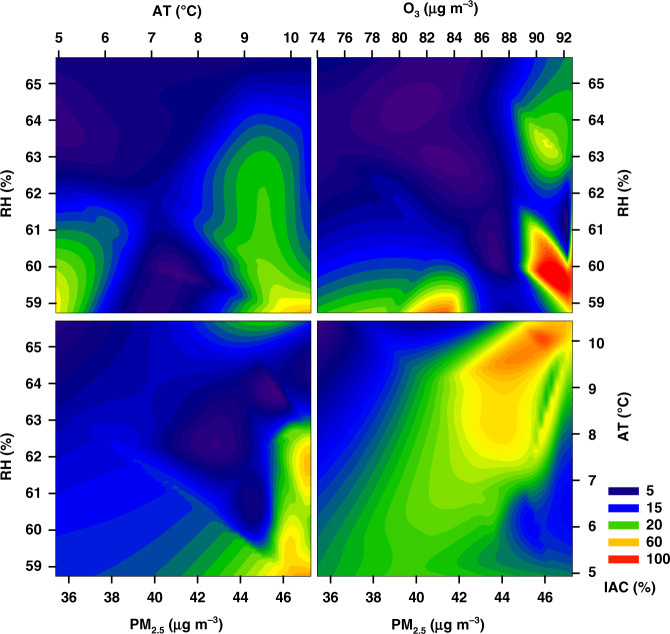
The joint relationships between IAC and multiple environmental factors (e.g., PM_2.5_, O_3_, Air temperature, and Relative humidity) in Northeast China. IAC incidence of allergic conjunctivitis, AT Air temperature, RH Relative humidity

For this study, we examined whether the prevalence of the IAC was significantly associated with environmental factors during 2014–2018 using the method of correlation coefficient analysis. According to paired monthly data of prefecture-level cities from 2014 to 2018, there seem to be no significant link among the IAC, the concentrations of atmospheric pollutants and the levels of meteorological factor. This may be due to the fact that the factors such as pollen released from plant growth are not fully taken into account in this study, which indirectly suggests that plant pollen is a more important factor for the intra-annual variation of the IAC. However, based on the annual provincial paired data from 2014 to 2018, a significant correlation is determined among most of these factors (Table 2). The IAC is significantly (*p* < 0.05) positively correlated with PM_2.5_, NO_2_, air pressure, and wind speed, with correlation coefficients of 0.57, 0.54, 0.63, and 0.54, respectively. Furthermore, the very significant (*p* < 0.01) positive correlation between the IAC and CO, SO_2_, PM_10_, O_3_, and air temperature are determined with correlation coefficients of 0.92, 0.85, 0.83, and 0.75, respectively. In contrast, there is a significant (*p* < 0.05) negative correlation (*r* = −0.61) between the IAC and relative humidity. Although both the meteorological factors and air pollution are significantly associated with the occurrence of AC, air pollution appears to play a more important role in the occurrence of AC than meteorological factors. Furthermore, given the importance of these factors (e.g., primary pollutants and major climate change indexes), four single factors (i.e., PM_2.5_, O_3_, temperature, and relative humidity) are screened for comprehensive correlation analysis (Fig. 4). With the increase of temperature and the decrease of humidity, the IAC appears to increase significantly, indicating dry and hot climate is likely to induce AC. Moreover, increased levels of air pollution, such as PM_2.5_ and O_3_, can significantly worsen the AC, especially in dry and hot regions.

**Table 2:** Correlation coefficients among the IAC, atmospheric pollutants (i.e., PM_2.5_, PM_10_, SO_2_, CO, NO_2_, and O_3_) and meteorological factors (i.e., Air pressure, Air temperature, Relative humidity, Visibility, Precipitation, Wind speed, and Wind direction)

	IAC	PM_2.5_	PM_10_	SO_2_	CO	NO_2_	O_3_	AP	AT	RH	V	PR	WS_1_	WS_2_	WD
IAC	1														
PM_2.5_	0.57^a^	1													
PM_10_	0.75^b^	0.94^b^	1												
SO_2_	0.85^b^	0.82^b^	0.88^b^	1											
CO	0.92^b^	0.79^b^	0.90^b^	0.98^b^	1										
NO_2_	0.54^a^	0.76^b^	0.64^a^	0.82^b^	0.72^b^	1									
O_3_	0.83^b^	0.62^a^	0.84^b^	0.76^b^	0.86^b^	0.29	1								
P	0.63^a^	0.29	0.32	0.72^b^	0.67^b^	0.71^b^	0.34	1							
T	0.74^b^	0.57^a^	0.71^b^	0.72^b^	0.78^b^	0.42	0.79^b^	0.41	1						
H	−0.61^a^	−0.35	−0.51	−0.43	−0.51	−0.13	−0.57^a^	−0.15	−0.12	1					
V	0.04	0.43	0.23	0.33	0.22	0.59^a^	−0.12	0.37	−0.22	−0.21	1				
PR	−0.41	0.13	−0.17	−0.09	−0.24	0.42	−0.62^a^	0.06	−0.45	0.37	0.71^b^	1			
WS_1_	0.54^a^	0.40	0.66^b^	0.38	0.53^a^	−0.20	0.84^b^	−0.14	0.74^b^	−0.26	−0.59^a^	−0.71^b^	1		
WS_2_	0.50	0.31	0.55^a^	0.25	0.41	−0.19	0.71^b^	−0.30	0.54^a^	−0.47	−0.49	−0.70^b^	0.94^b^	1	
WD	0.04	−0.15	−0.14	−0.01	−0.01	0.08	−0.17	0.12	0.00	−0.08	0.19	0.10	−0.19	−0.02	1

IAC incidence of allergic conjunctivitis, AP Air pressure, AT Air temperature, RH Relative humidity, V Visibility, PR Precipitation, WS_1_ and WS_2_ represent wind speed in the height of 2m and 10 m, respectively.

The symbol of ^a^and ^b^indicate the significant (*p* < 0.05) and very significant (*p* < 0.01) correlations, respectively.

### Threshold of environmental factors for allergic conjunctivitis

**Fig. 5:**
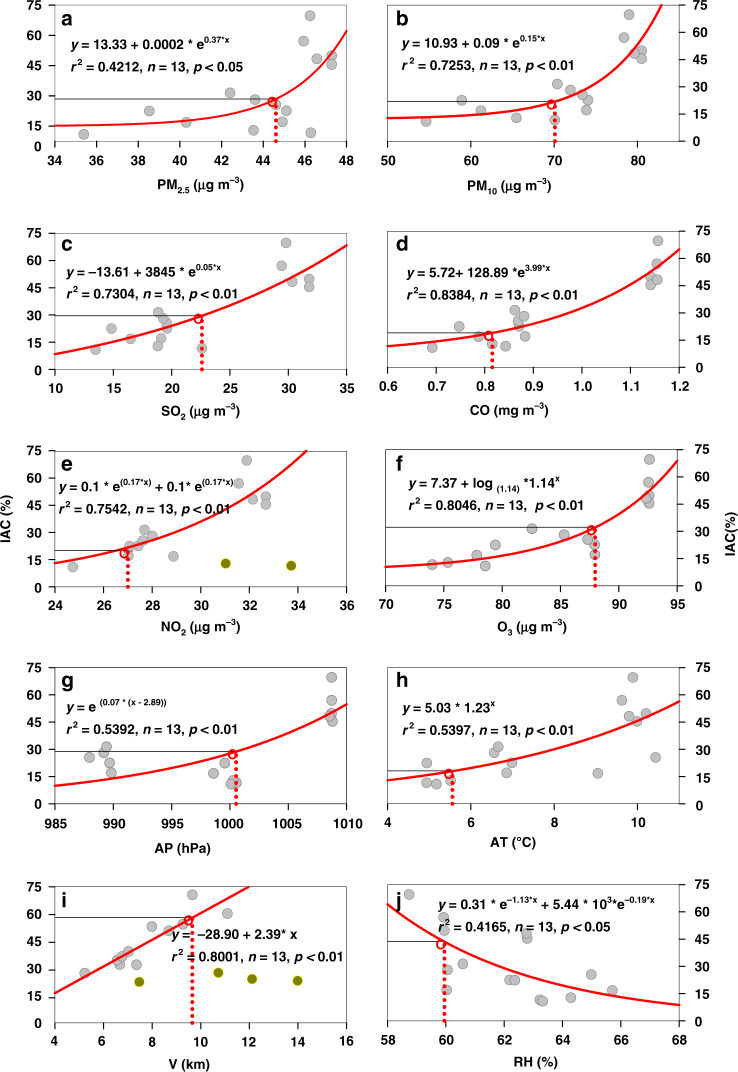
The linear or nonlinear regression curves between IAC and each environmental factors and their corresponding threshold values (i.e., PM_10_, PM_2.5_, SO_2_, CO, NO_2_, O_3_, Air pressure, Air temperature, Visibility, and Relative humidity) in Northeast China. IAC incidence of allergic conjunctivitis, AP Air pressure, AT Air temperature, RH Relative humidity, V Visibility, PR Precipitation. The gray solid circle is the paired data for the IAC and each factor; the yellow solid circle is outliers that not used in regression analysis; the red hollow circle is the threshold for each factor

The results above-mentioned show that AC is associated with environmental factors, and each pollutant or meteorological factor may influence the prevalence of AC. We estimated the contribution of each atmospheric pollutant and meteorological factor to the prevalence of AC using nonlinear regression analysis (Fig. 5), and further qualified their critical threshold points of environmental factors for AC diseases using threshold regression modeling (TRM) analysis (Table 3). Consistent with the *r* values calculated using correlation analysis in Table 1, significant associations were observed between the prevalence of AC and the level of atmospheric pollutants. The meteorological factors with weak influence are omitted from the analysis and are summarized for representative meteorological conditions. Threshold analysis is to determine the value of the parameter at the break-even point. The threshold values of six atmospheric pollutants for the AC were 23 μg m^−3^ in SO_2_, 27 μg m^−3^ in NO_2_, 0.82 mg m^−3^ in CO, 88 μg m^−3^ in O_3_, 70 μg m^−3^ in PM_10_, and 45 μg m^−3^ in PM_2.5_, respectively (Table 3). For air quality, different countries, regions, and organizations have greatly different standards. WHO, on the basis of setting air quality standards, proposed two transition periods, with Goal 1 and Goal 2; China’s Air Quality Standards divides standards into two Classes, with Class 1 for special regions (including national parks) and Class 2 for all other regions (Table 3). Our results show that the values of thresholds of PM_10_ (70 μg m^−3^) and PM_2.5_ (45 μg m^−3^) are higher than China’s national air quality standard and the standard advised of the WHO for the IAC. The SO_2_ threshold of 23 μg m^−3^ is comparable to the WHO advised standard but significantly below China’s criterion, indicating bigger environmental risks in China. The thresholds of NO_2_ (27 μg m^−3^) and O_3_ (88 μg m^−3^) are both significantly lower than current standards, attributable to major environmental risk factors for the IAC. For meteorological factors, the threshold values for the most important indexes of air temperature, relative humidity were 5.5 °C and 60%, respectively. The threshold points of air temperature are at the end of the range in Northeast China, suggesting a high IAC, but at the same time, the relative humidity threshold is also at the lower end, which means the risk potential of AC is low. Thus, climate-induced AC should vary greatly from city to city due to natural conditions and urban management and planning.

**Table 3:** The threshold of environmental factors for the IAC and corresponding criteria in China and WHO

Item	Index	Threshold of allergic conjunctivitis	China standard		WHO transition		WHO standard
			Class 1	Class 2	Goal 1	Goal 2	
Air pollutant	SO_2_ (μg m^−3^)	23	20	60	70	–	20
	NO_2_ (μg m^−3^)	27	40	80	40	40	40
	CO (mg m^−3^)	0.82	4	4	–	–	–
	O_3_ (μg m^−3^)	88	100	100	160	–	100
	PM_10_ (μg m^−3^)	70	40	70	70	50	20
	PM_2.5_ (μg m^−3^)	45	15	35	35	25	10
Meteorological Factors			Annual	Max	Min	Standard deviation	
	AT (°C)	5.5	7.9	10.0	5.0	2.1	
	RH (%)	59.9	62.1	65.0	59.0	2.1	
	AP (hPa)	1000.0	999.0	1009.0	988.0	8.4	
	V (km)	9.7	8.7	13.9	5.2	2.4	
	PR (mm)	783.2	882.0	2840.0	473.0	778.8	

IAC incidence of allergic conjunctivitis, WHO World Health Organization, AT Air temperature, RH Relative humidity, AP Air pressure, V Visibility, PR Precipitation.

O_3_ concentration stand for annual average max 8 h data; CO concentration threshold of AC stand for the annual average data, and the China Standard represents the daily average data; China Air Quality Standards divides into Class 1 (special regions) and Class 2 (all other areas); WHO not only establishes guideline values but also establishes two transition standards (i.e., Goal 1 and Goal 2).

## Discussion

The prevalence of AC has been increasing in a rapid fashion in recent years. The exact prevalence of AC varies in different countries or even in different regions of the same country. Convincing evidence demonstrate that both air pollution and meteorological variables can cause AC. Our study, for the first time, demonstrates that both atmospheric pollutants and meteorological factors are significantly associated with AC in Northeast China, and the former plays a more important role. Our results show that atmospheric pollutants (i.e., PM_2.5_, PM_10_, SO_2_, NO_2_, CO, and O_3_) and meteorological variables (e.g., high temperature and low humidity) are positively correlated with AC, indicating that these factors may promote the onset or aggravate AC. In addition, we established, for the first time, thresholds of important environmental factors included atmospheric pollutants and meteorological factors for AC, which can be of great significance for public care and local regional atmospheric environment management and planning.

PM_2.5_ mainly includes organic compounds, metallic, and combustion particles. PM_10_ mainly includes molds, dust, and pollens. The significant association between PMs and AC has been well documented. Previous studies have shown that higher levels of PMs are associated with an increase in outpatient visits for AC. The pathogenesis of PMs (i.e., PM_2.5_, PM_10_, and DEP) induced-AC is multifaceted. And the underlying mechanisms are partially elucidated. In the phase of AC, the allergen firstly contacts with the conjunctiva, and then it is processed and presented by antigen-presenting cells and/or dendritic cells, causing helper T cells or naive CD4 cells mature, sensitization and differentiate into Th2 cells^15^. Th2 cells participate IgE-mediated allergies by the way of inflammatory cytokine releasing (e.g. IL-13, IL-10, IL-9, IL-5, IL-4, and IL-3)^15^. These allergies and B cells produce IgE, mast cell growth, and aggregation of eosinophil cells^16^. Eosinophil cells release histamine expression and induce conjunctival allergic reaction, such as hyperemia, microvascular permeability, cytokine secretion, etc^17^. It has been reported that PM_2.5_ triggers AC by inducing serum IgE. PM_2.5_ has also been found to induce mast cells and eosinophilic cells infiltration into the conjunctiva in a murine model. The mast cell is known as a key immune-regulatory factor in the Th2-mediated responses, indicating PM_2.5_ might trigger Th2-dominant ocular immune response^18^. In a study conducted in Tokyo, elevated level of atmosphere PM_2.5_ was found to be considerably associated with an increase in numbers of outpatient visits (*r* = 0.62, *p* = 0.018) for AC from May to July, while there seems to be no statistical significance of correlation from August to November, indicating that PM_2.5_ may have played a role in AC development during the nonpollen season^19^. The prevalence and symptoms of AC are significantly associated with levels of PM_10_ (odds ratio = 1.54) in Japan^20^.

In our study, we find that both PM_2.5_ (*r*^2^ = 0.570, *p* < 0.05) and PM_10_ (*r*^2^ = 0.748, *p* < 0.01) are positively correlated with AC, which has shown a similar tendency comparable with the Tokyo study. However, a retrospective registry analysis in Hong’s study finds that there seems to be no association between PM_s_ and AC in Shanghai, China^21^. The differences between the two studies may be due on the one hand to a discrepancy in research scales, data sources, and statistical methods; and on the other hand, to dissimilarities in the sources of air pollution at each region, particulate matter is the primary pollutant in Northeast China, but it is not necessarily the primary pollutant in megacities of East China.

In our study, AC is proven to be positively correlated with both SO_2_ (*r*^2^ = 0.849, *p* < 0.01), O_3_ (*r*^2^ = 0.832, *p* < 0.01), and CO at (*r*^2^ = 0.916, *p* < 0.01), indicating SO_2_, O_3_, and CO may trigger the onset or aggravates AC. It is also proven in Shanghai that elevated atmosphere concentration of O_3_ and NO_2_ are strongly correlated with increased outpatient visits for AC^21^, which is coherent with our study. SO_2,_ O_3_, NO_2_ may increase allergic sensitization response^20,22^, and also promote conjunctival inflammation via direct oxidative damage to the ocular surface and acidification of the tears, leading to AC. NO_2_ and O_3_ may also induce conjunctival inflammation indirectly via chemical modifications of aeroallergens and subsequent enhanced allergic response^12^. The ocular surface can be directly damaged by these pollutants as they can reduce the pH of the lacrimal fluid or oxidization. These pollutants promote allergen penetration, induce inflammation and promote cytokines release, resulting in damages to the mucosal surface^23^. In addition, CO, another important indicator of AC, is mainly produced in the process of energy consumption, vehicle emissions, and inadequate burning of crop straw in Northeast China, alludes to the diversity of sources of atmospheric pollutants that affect AC.

Our results suggest that the IAC may increase with regional climate changes in Northeast China, such as warming and drying. Climate change may increase the frequency, intensity, and duration of extreme weather events (e.g., heat waves, floods, bushfires, and storms), which poses significant and direct risks to human health^24^. Climatic conditions (temperature, wind speed, relative humidity, etc.) can affect AC. Previous studies have indicated that Northeast China is one of the area most susceptible to climate change, which has already seen an average temperature increment of 0.38 °C per decade during the past five decades^25^. It has also been reported that higher temperature is strongly correlated with increased outpatient visits for AC in Shanghai, China^21^. The daily mean temperature reported is correlated with other meteorological factors (relative humidity, sunshine, and wind speed) in the occurrence of allergic diseases^26^. Higher temperature may stimulate earlier flowering, leading to longer pollen seasons^27^. Elevated levels of NO_2_ and O_3_ are significantly related to an increased prevalence of AC, especially in warm conditions^12^. Strong wind and low humidity provoke evaporation of the land surface, leading to increased concentration of airborne pollens and air pollutants^28^. High temperature, strong wind, and pollens working together can aggravate conditions resulting in decreased stability of the ocular surface^20^. A nationwide survey was conducted in Japan and the results show that the prevalence of AC is as high as 48.7% in Japan, mostly caused by cypress and cedar pollen^20^.

A similar trend is observed for AC in our study, the IAC is mainly concentrated in July to September, with the peak occurring in August. Temperature and wind speed are positively correlated with AC (*r*^2^ = 0.739, *p* < 0.01; *r*^2^ = 0.539, *p* < 0.05), but relative humidity is negatively correlated (*r*^2^ = −0.608, *p* < 0.05). Meteorological factors such as temperature and relative humidity can affect the physiology, distribution, and amounts of these allergens^29^. Pollens are considered a well-known allergy trigger and it has been reported that higher pollen-specific IgE is found in tears of AC patients than those of controlled subjects^22^, while pollen-Tear-specific IgE in AC patients reveals negative results in serum, indicating that local allergic sensitization response is activated and the conjunctiva might have contributed to the synthesis of local IgE^30^. Increased number of double-positive cells (FceRI + CD1a +) in AC patients than in nonatopic controls suggest that increased IgE receptor expression facilitates the role of antigen presentation^31^.

In this study, we find that, in general, higher temperatures, low relative humidity, high level of surrounding O_3_, and atmospheric particles (PM_2.5_), are all great risk factors for the development as well as the progression of AC. Therefore, exposure to air pollution may have severe impacts on ocular health, especially in Northeast China. Studying the relationship between the IAC and regional environment is one of the most important research directions in order to solve the pathogenesis of AC in specific regions.

During 2013–2017, with the strengthening of air pollution control in China, air quality in various Chinese regions improved significantly with a downward trend for the concentrations of most atmospheric pollutants (i.e., PM_2.5_, PM_10_, SO_2_, NO_2_, and CO), while the concentration of O_3_ demonstrated an upward trend at the same period^32^. Atmospheric pollutants have similar interannual variation characteristics in Northeast China^13^. After 2018, as a result of synergistic emission reduction control measures (e.g., cleaner transport, energy-efficient homes, power generation, industry, and better municipal waste management) of atmospheric PMs and O_3_ implemented in China, both the PMs and O_3_ concentrations decreased, and the average annual O_3_ concentration decreased by 5.0% in Northeast China. The trend also suggests a reduced risk of AC in the following years in Northeast China. However, there is still a great uncertainty concerning this change, which requires a comprehensive combination study of the outpatient survey, medical care system, detailed atmospheric pollutant components, urban, or regional plant species. It is necessary for future research to evaluate the relationship between the social, demographic, agricultural, regional, and economic factors and AC. Additionally, the analysis of the relationship between AC and other direct or indirect factors is of great significance to public health, economic development, and relevant environmental policy formulation.

The IAC data in the previous studies are usually obtained from the ophthalmic examinations or questionnaire on the subjects involved. In this study, because it is difficult to obtain the number of patients for AC directly and simultaneously at different cities in Northeast China, we used an indirect method to obtain the estimated data of IAC in each city based on the sales of the main therapeutic antiallergic drugs for the treatment of AC and normalized by the proportion at the municipal level. Although this method is a well-established one and has been used previously in the estimation of the prevalence of various diseases including AC^33–38^. However, since the data was not collected from outpatient clinics, there was no detailed patient information in this study. The sales of these medications are influenced by the economic and medical levels of the residents. Therefore, this is a comparative data that should be used carefully in the comparison with the data obtained from the direct methods.

The occurrence of allergic disease is related to environmental factors, biological factors, and exposure level of population^4,26^. At the interannual scale, when biological factors and exposure levels are excluded, air pollution is shown to be the major influencing factor. Nevertheless, most of the environmental factors have no significant correlation with AC on seasonal and monthly scales in our study. The IAC was high in summer months with the peak in August, possibly due to an increased risk of allergen exposure during the summer holidays and relatively high levels of biological factors, such as pollen release. Pollen is a critical trigger for many allergy and asthma sufferers, and pollen concentrations are strongly linked to drug sales^39^. Thus, the relationships between pollen and AC in Northeast China need to be further studied.

**Fig. 6:**
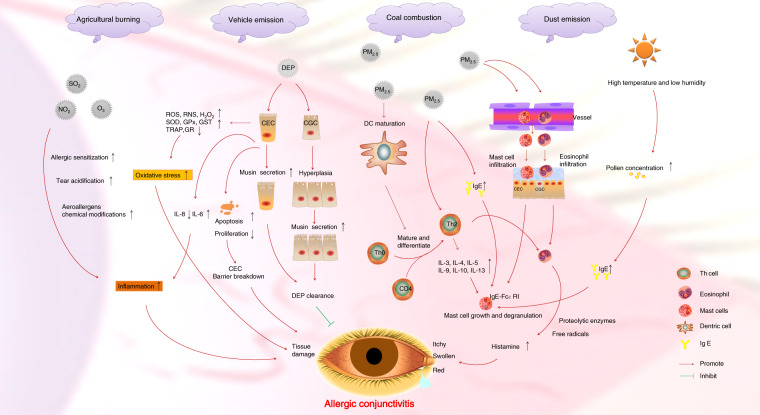
The effects and mechanism of air pollution and meteorological factors on AC in Northeast China. NO_2_, SO_2_, O_3_ increase allergic sensitization, induce acidification of the tears, and chemical modification of aeroallergens. High temperature and low humidity increase pollen concentration. Pollens induce IgE production and IgE attached to the IgE-FcεRI complex on mast cells (MGs), activating MGs degradation and releasing histamine. PM_2.5_ promotes IgE production, MGs growth, and aggregation of eosinophil cells infiltration in the conjunctiva. Allergens were presented by dendritic cells (DC), causing Th0 or CD4 cells to differentiate into Th2 cells, promoting IgE-mediated allergies by releasing IL-3, IL-4, IL-5, IL-5, IL- 9, IL-10, and IL-13, leading to eosinophilic activation and releasing histamine. Diesel Exhaust Particles (DEP) decreased the viability, proliferation, IL-8 expression and increases apoptosis, and IL-6 expression in conjunctiva epithelial cells (CEC). DEP induces conjunctiva goblet cells (CGC) hyperplasia and secrete musin to facilitate DEP clearance. DEP increases the expression of ROS, nitrogen (RNS), hydrogen peroxide (H_2_O_2_), superoxide dismutase (SOD), glutathione peroxidase (GPx), and glutathione S-transferase (GST). DEP decreases the expression of glutathione reductase (GR) and the total reactive antioxidant potential (TRAP) in CEC, resulting in AC

This study, for the first time, elucidates the association between the IAC, air pollution, and weather changes in Northeast China. It is a multidisciplinary crossing study conducted in 20 cities in the region over 5 years. We find that the IAC is affected both by air pollution and climate change, while air pollution plays a more important role in the incidence of disease at the regional level. Worsening air quality and a hotter, drier climate are likely to trigger more cases of AC. The thresholds of annual atmospheric pollutants (PM_10_: 70 μg m^−3^; PM_2.5_: μg m^−3^; SO_2_: 23 μg m^−3^; NO_2_: 27 μg m^−3^; O_3_: 88 μg m^−3^; CO: 0.82 mg m^−3^) and meteorological factors (air temperature: 5.5 °C; relative humidity: 60%) that trigger more AC are quantified, providing key parameters for measures and standard setting in urban air pollution control. The effects and mechanism of air pollution and meteorological factors on AC in Northeast China is summarized (Fig. 6). These findings suggest the potential for a gradual reduction in the IAC in the future with the increase in coordinated control of air pollution and climate change in China.

## Materials and methods

### Study area

**Fig. 7:**
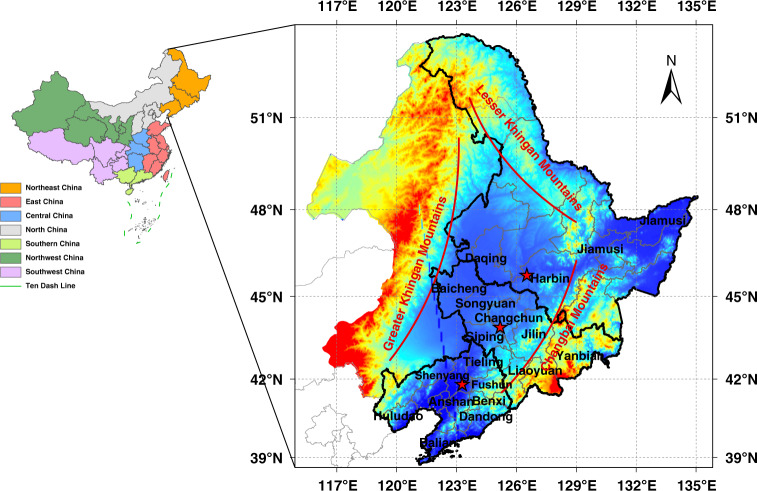
Topography, administrative division, and cities with data sources in the three provinces of Northeast China. Tagged cities represent the valid data of both air pollution and allergic conjunctivitis

Northeast China includes the three provinces of Liaoning, Jilin, and Heilongjiang, which have a total population of 110 million and a land area of 787,300 square kilometers (Fig. 7). The heartland of the region is the Northeast China Plain, which is a major breadbasket region with a total area of 3.5 × 10^5^ km^2^. The main crops are single-season crops such as corn, soybean, rice, and wheat, with the growing season usually May to September. In the past decade, crop straw is often burned in the open air, aggravating seasonal air pollution. Northeast China has a temperate monsoonal climate with four distinct seasons, of which winter is cold and long, lasting 4–6 months, with a minimum monthly mean temperature of −12 °C to −19 °C. Such a frigid climate means that the burning of fuels for heating, including coal, biomass, and natural gas, releases far more atmospheric pollutants than elsewhere. At present, Northeast China is still a heavy industrial base for China’s steel, petroleum, petrochemical, shipbuilding, machine tool, aviation, and automotive industries. However, most of these heavy industries belong to traditional high-energy consumption, high-pollution, and resource-dependent enterprises, which is one of the important causes of air pollution. In addition, Northeast China is one of the regions with the most intense global climate change due to its high latitude.

### Study roadmap

**Fig. 8:**
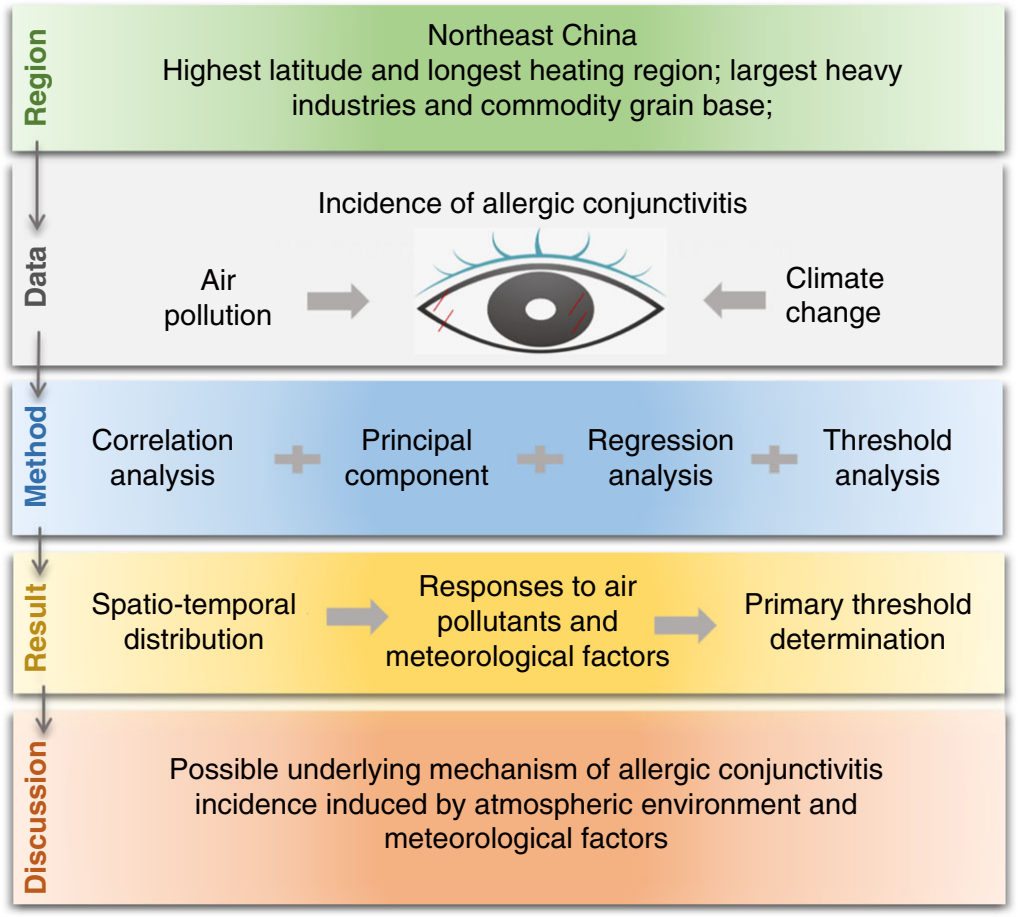
Roadmap of research technology for the meteorological factors and atmospheric pollutants effect to the IAC in Northeast China. IAC incidence of allergic conjunctivitis

Compared with other regions of China, Northeast China has the highest latitude, the longest central heating period, the largest heavy industries base, and the largest commodity grain base, which result in quite unique climatic and air pollution factors. We started the study from the hypothesis that the prevalence of the IAC in the region is significantly associated with the air pollution the meteorological conditions in Northeast China. First, we collected the daily average data of six standard air pollutants and meteorological factors, as well as valid monthly data of the IAC at the scale of prefecture-level cities, and analyzed the temporal and spatial trends of this information in detail. Then, based on these data, the relationships between the IAC and air pollutants and meteorological factors were established, the main influencing factors were screened out, and the threshold values of the main influencing factors was obtained using the analysis methods of correlation, principal components, regression, and threshold. Finally, the responses of IAC to air pollution and climate change, possible underlying mechanism, and future environmental risks are proposed (Fig. 8).

### Data Sources

Data sources are mainly composed of IAC data, air quality, meteorological conditions, and social and economic indicators, which can help with a comprehensive evaluation the correlation between the IAC and air pollution, climate changes, and social and economic values.

### Incidence of allergic conjunctivitis

Estimating of the prevalence of a particular disease based on drug sales (data from manufactory, stores, or health insurance) have been reported previously^33–38^. Since it is difficult to obtain the number of patients for AC directly and simultaneously at different cities in Northeast China, we take an indirect way to get the estimated data of IAC in each city based on the sales of the main therapeutic drugs for AC and normalized by the proportion at the municipal level. Santen Pharmaceutical Co., Ltd. and Alcon Laboratories Inc. (a Novartis company), two leading enterprises in the ophthalmic prescription drug market, provide drugs with typical pertinence (drug pertinence credibility of more than 90%) for the treatment of AC as test indicators in Northeast China. The drugs are 0.1% Pemirolast Potassium Eye drops (Santen Pharmaceutical Co., Ltd, Japan) and 0.1% Olopatadine Hydrochloride Eye Drops (S. A. Alcon-Couvreur N. V., Belgia), respectively.

### Air quality and meteorological factors

Daily urban air quality index (AQI) and six atmospheric pollutants (i.e., PM_10_, PM_2.5_, SO_2_, NO_2_, CO, and O_3_) of prefecture-level cities in Northeast China between 2014 and 2018 are obtained from China National Environmental Monitoring Center. Contemporaneous ground meteorological indexes include air temperature, air pressure, precipitation, relative humidity, visibility, wind speed, and wind direction of cities included in the study are collected from China Meteorological Administration. For the indexes of air quality and meteorology, daily data were processed into monthly, seasonal and annual scales. Among all indexes, only the precipitation data is of cumulative value, other indicators are the average value of the corresponding time period.

### Monitoring method

The data of air pollution and meteorological factors in cities are monitored by national standard instruments, monitoring standards, calibration methods, and unified operation and maintenance management to ensure the accuracy of data in China. Based on the technical standard for automatic monitoring of air quality in China (HJ 193–2016), the real-time concentrations of NO_2_, SO_2_, O_3_, CO, and particulate matter (i.e., PM_10_ and PM_2.5_) were determined by the methods of chemiluminescence, ultraviolet fluorescence, ultraviolet photometry, gas filtering correlation infrared absorption, and β-ray, respectively. According to the Chinese Meteorological Observation Standard (GB/T 33703-2017), the indexes of air temperature, relative humidity, precipitation, air pressure, wind speed, and direction were monitored in real-time by automatic meteorological observation instruments. Visibility data was measured by an automatic visibility meter, which was composed of an optical transmitter, optical receiver, and microprocessor controller.

### Statistical analysis

IAC data, indexes of air quality, and meteorology of prefecture-level cities were matched at the same time scale for further analysis. Pearson correlation analysis and regression analysis among all indexes were conducted to qualitatively and quantitatively estimate the associations of IAC and environmental factors. The significance of the differences in the atmospheric pollutant concentrations and meteorological indexes were determined using the independent-samples *T* test. The analysis of principal component (PCA) and threshold were achieved to clarify the dominant factors and corresponding threshold inflection points, which are important information for further healthy assessment and control goal of air pollution. All analysis and plotting were performed using the R program and related packages.
